# SARS-CoV-2 epidemic after social and economic reopening in three U.S. states reveals shifts in age structure and clinical characteristics

**DOI:** 10.1126/sciadv.abf9868

**Published:** 2022-01-26

**Authors:** Nathan B. Wikle, Thu Nguyen-Anh Tran, Bethany Gentilesco, Scott M. Leighow, Emmy Albert, Emily R. Strong, Karel Brinda, Haider Inam, Fuhan Yang, Sajid Hossain, Philip Chan, William P. Hanage, Maria Messick, Justin R. Pritchard, Ephraim M. Hanks, Maciej F. Boni

**Affiliations:** 1 Center for Infectious Disease Dynamics, Department of Statistics, Pennsylvania State University, University Park, PA, USA.; 2 Center for Infectious Disease Dynamics, Department of Biology, Pennsylvania State University, University Park, PA, USA.; 3 Department of Medicine, Brown University, Providence, RI, USA.; 4 Center for Infectious Disease Dynamics, Department of Bioengineering, Pennsylvania State University, University Park, PA, USA.; 5 Department of Physics, Pennsylvania State University, University Park, PA, USA.; 6 Center for Communicable Disease Dynamic, Department of Epidemiology, Harvard T.H. Chan School of Public Health, Boston, MA, USA.; 7 Department of Biomedical Informatics, Harvard Medical School, Boston, MA, USA.; 8 Yale School of Medicine, Yale University, New Haven, CT, USA.; 9 Rhode Island Office of the Governor and Rhode Island Department of Health, Providence, RI, USA.; 10 Nuffield Department of Medicine, University of Oxford, Oxford, UK.

## Abstract

State-level reopenings in late spring 2020 facilitated the resurgence of severe acute respiratory syndrome coronavirus 2 transmission. Here, we analyze age-structured case, hospitalization, and death time series from three states—Rhode Island, Massachusetts, and Pennsylvania—that had successful reopenings in May 2020 without summer waves of infection. Using 11 daily data streams, we show that from spring to summer, the epidemic shifted from an older to a younger age profile and that elderly individuals were less able to reduce contacts during the lockdown period when compared to younger individuals. Clinical case management improved from spring to summer, resulting in fewer critical care admissions and lower infection fatality rate. Attack rate estimates through 31 August 2020 are 6.2% [95% credible interval (CI), 5.7 to 6.8%] of the total population infected for Rhode Island, 6.7% (95% CI, 5.4 to 7.6%) in Massachusetts, and 2.7% (95% CI, 2.5 to 3.1%) in Pennsylvania.

## Teaser

COVID-19 clinical outcomes improved over the first 6 months, but infection fatality rates were initially above 1.5%.

## INTRODUCTION

The coronavirus SARS-CoV-2 (severe acute respiratory syndrome coronavirus 2), the cause of coronavirus disease 2019 (COVID-19), has led to more than 750,000 deaths across the United States in just 20 months of transmission. During the initial wave in spring 2020, a critical question in managing the U.S. COVID-19 epidemic was whether regional reopenings of social and economic activity would result in rebounds of cases and hospitalizations (*1*). Because population-level immunity to SARS-CoV-2 was still low at the time, the expectation was that increases in mobility and human contact would lead back to an upward trending epidemic curve during this time (*2*). However, as hand hygiene, physical distancing, epidemiological awareness, and mask-wearing practices changed during spring 2020, increases in daily economic and social activity were not guaranteed to recreate the ideal transmission conditions of March. In addition, no school session and the possible effects of drier/hotter weather (*3*) in summer were considered potential mitigators on viral transmission (*4*).

Despite these mitigating factors, epidemiological rebounds had begun in more than 40 states by 1 July. Daily case numbers in the United States, which had dropped from a peak of 30,000 per day in early April to 20,000 per day in late May, rebounded to 50,000 per day the first week of July (*5*, *6*) driven by early reopening policies in several large states. With a symptomatic case fatality rate (sCFR) in the 1 to 4% range (*7*–*11*) depending on epidemiological context and testing availability, more than a thousand of these daily new case numbers would result in death several weeks later. The absence of careful, gradual, managed reopenings during the May/June period was the likely cause of summer resurgence in parts of the southern United States. It is of utmost public health importance that epidemic management and public health response continues to be approached with a strategic and adaptive plan that can use real-time epidemiological analysis (e.g., attack rate estimates, changing age/mobility patterns, and clinical improvements) to contain and potentially reverse upward epidemic trends.

Here, we analyze the age-structured case, hospitalization, and death time series from three states—Rhode Island (RI), Massachusetts (MA), and Pennsylvania (PA)—that, during summer 2020, did not experience substantial epidemic rebounds when compared to March/April levels. We evaluate 11 clinical data streams reported by the respective state health departments in a Bayesian inference framework built on an ordinary differential equation (ODE) age-structured epidemic model that includes compartments (clinical states) for hospitalization, critical care, and mechanical ventilation. We infer parameters on surveillance, transmission, and clinical characteristics of the first epidemic wave in RI, MA, and PA. We describe the patterns of persistently low transmission in these three states throughout 31 August, compare these low-transmission scenarios to changes in human mobility metrics, and evaluate changes in age structure and clinical outcomes. We evaluate the impact of the spring epidemic on elderly populations in these three states, and we compare infection fatality rates (IFRs) to published estimates from other parts of the world. Preliminary analyses resulting from this work were regularly posted at https://mol.ax/covid and shared with the respective state departments of health (DOHs). The statistical inference described here—on attack rates, underreporting, and changing age profiles—can guide more granular real-time decision-making and public health messaging than data streams alone.

## RESULTS

### Epidemic characteristics during and after lockdown

**Fig. 1.**
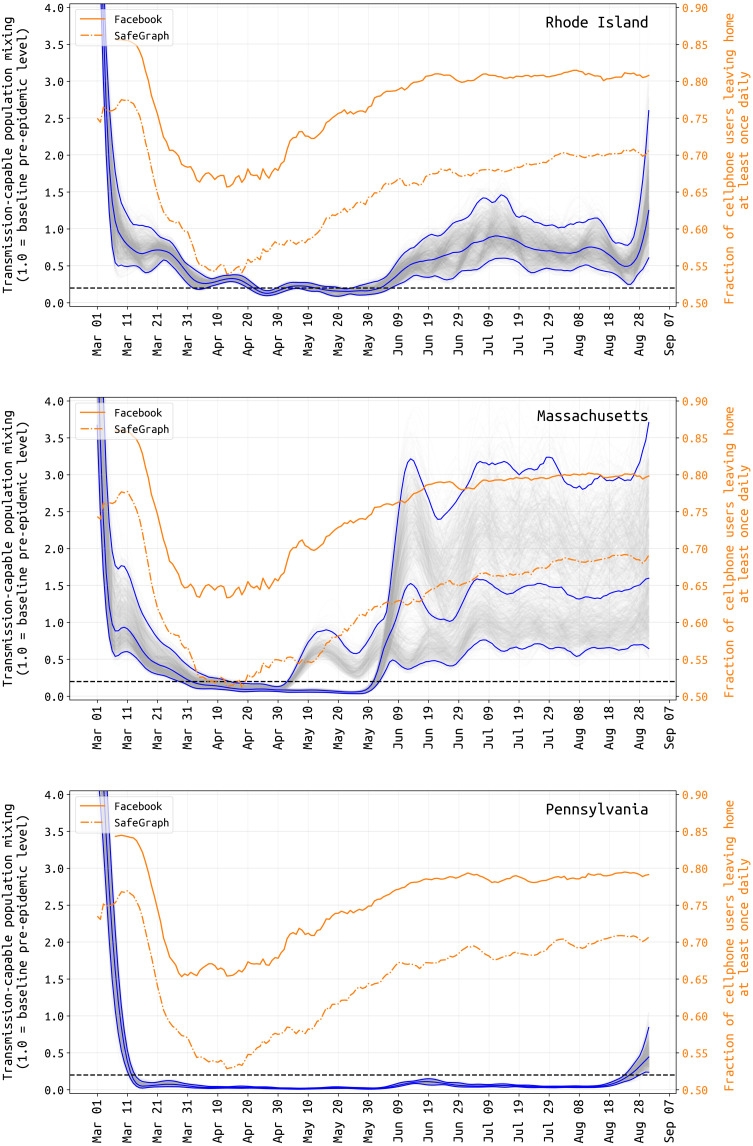
Comparison of estimated transmission-capable population-level mixing with population mobility. Transmission-capable population-level mixing β_t_ (in gray and blue) and mobility changes (orange) from 1 March to 31 August. The average population mixing for 5 to 15 March is set to 1.0 as the pre-epidemic level of transmission-capable mixing, and all other values are reported relative to this. Gray lines show 1000 sampled posterior β-trajectories with the blue lines showing the median and 95% CIs. Note that there is substantial uncertainty in these estimates during the first weeks of March, as case numbers were low and reporting may not have been catching a large proportion of true cases at this time. Orange lines show the fraction of Facebook and SafeGraph users that left home at least once per day. The correlation between population movement (orange) and transmission-capable population movement (gray and blue) begins to disappear in early May in RI and PA (and with less certainty, in MA).

In RI, MA, and PA, from early March to early April, we inferred a reduction in the composite transmission parameter β_t_ describing person-to-person contact (mixing) rates and the probability of virus transmission per unit contact. From the 5 to 15 March period to 1 April, population mixing rates dropped by 65.2% [95% credible interval (CI), 51.4 to 78.2%] in RI, by 79.8% (95% CI, 61.5 to 87.0%) in MA, and by 95.4% (95% CI, 92.8 to 97.4%) in PA (Fig. 1). During this period, contact rates were dropping through stay-at-home orders, bans on large gatherings, and business/school closures at the same time as improved hygiene behaviors were being increasingly adopted. As we do not model the timings and effects of these individual interventions, we do not attribute their separate contributions [e.g., see Zimmerman and Anderson (*12*)] to lower estimates of β_t_. We refer to reductions seen in β_t_ in March and early April as reductions in transmission-capable mixing that result both from fewer person-to-person contacts and lower infection risk per contact. The estimated reductions in mixing may seem very large, but note that in a heterogeneously exposed population, mixing rates for large highly connected groups can drop by large amounts with only a modest drop in the population’s effective reproduction number *R*_t_ if a smaller subpopulation maintains a chain of infection due to an inability to completely zero-out contacts. For example, if 90,000 office employees can work from home and contact only their families but 10,000 elderly care home residents still require contact with medical and care staff, then a full business shutdown may result in a 90% reduction in mixing patterns but a measured or apparent *R*_t_ ≈ 1 if a stable chain of infection is maintained in nursing homes and elderly care residences. Our estimated reductions in transmission-capable mixing are consistent with published estimates of changes in *R*_t_ and mobility (*13*–*17*).

Changes in the inferred population-mixing component β_t_ can be compared to mobility metrics (*18*, *19*) on the basis of location-enabled smartphone data trails, which allow calculation of time spent at home versus outside the home. Two independent mobility data sources, Facebook and SafeGraph (*20*, *21*), provided daily estimates for the fraction of tracked users leaving home at least once in a 24-hour period. Despite values varying slightly across states and markedly between user bases, all mobility data examined have a common shape and timing: an initial baseline in early March (84 to 86% of users leaving home for Facebook and 75 to 77% for SafeGraph) and a subsequent marked decrease from 15 March to 31 March (64 to 67% for Facebook and 52 to 55% for SafeGraph). This low fraction of users leaving home (at a minimum, once daily) is maintained until about 20 April, followed by a slow increase to a slightly more cautious “new normal” (77 to 81% for Facebook and 66 to 69% for SafeGraph) through July and August (Fig. 1). A resumption of population mobility in early May suggests that improvements in hygiene, personal distancing, mask wearing, selective travel, and/or smaller event sizes were likely factors keeping *R*_t_ < 1 and new case numbers declining.

**Fig. 2.**
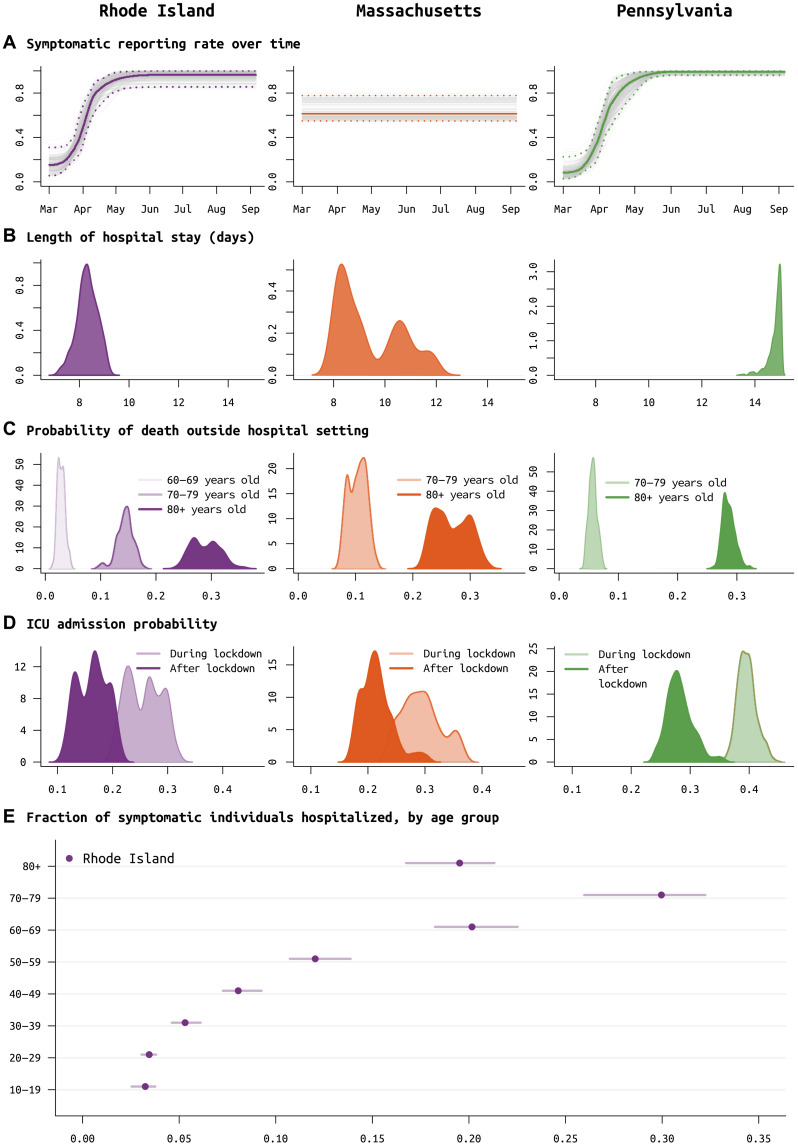
Posterior distributions of reporting rate and clinical parameters for RI, MA, and PA. (**A**) Reporting parameter ρ, i.e., the fraction of symptomatic SARS-CoV-2 cases that are reported to the health system, plotted as a function of time. In RI, it was known that in March, testing was not available and cases could not be confirmed; therefore, a spline function was fit for ρ. This same function provided a better fit for PA data, but not for MA data. (**B**) Median length of medical-floor hospital stay was 8.3 days in RI (purple, left column), 8.9 days in MA (orange, middle column), and 14.9 days in PA (green, right column). (**C**) Probabilities of death outside the hospital for the 60 to 69, 70 to 79, and 80+ age groups; 60 to 69 age group was included only for RI as data were insufficient in PA and MA. These are largely reflective of the epidemics passing through nursing home populations where individuals are not counted as hospitalized if they remain in care at their congregate care facility in a severe clinical state. These probabilities are important when accounting for hospital bed capacity in forecasts. (**D**) Age-adjusted ICU admission probability during the lockdown period in spring 2020 (lighter color) and after the lockdown (darker color). (**E**) Probability of hospitalization (median and 95% CIs) for symptomatic SARS-CoV-2 infections, by age group; estimates only available for RI.

**Fig. 3.**
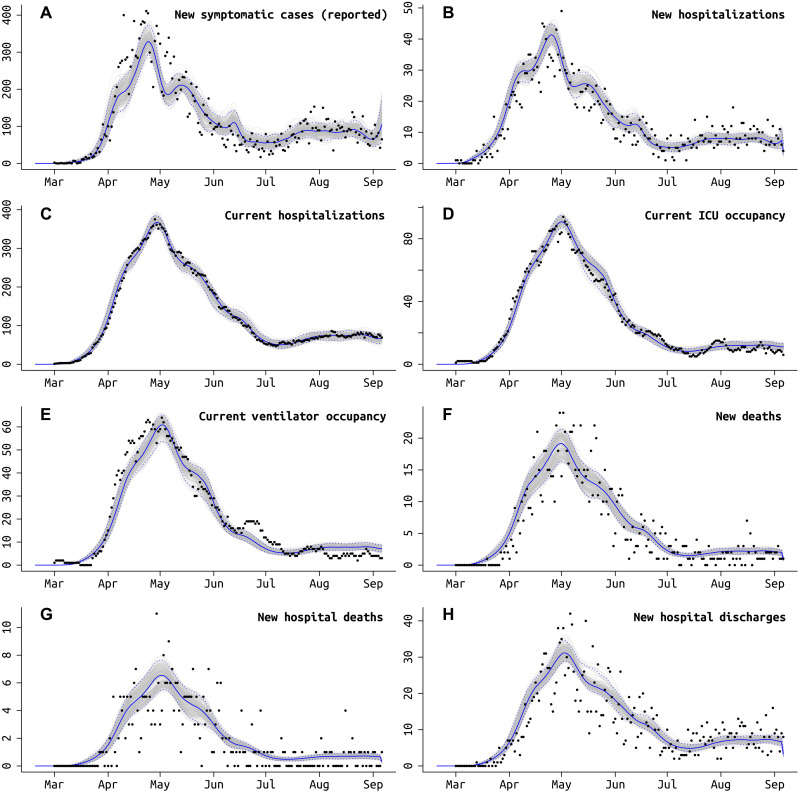
Model fit to RI daily data, using the best fit model that accounts for different age-based contact rates after the lockdown and a different rate of ICU admissions starting in early June (Table 2). Gray lines show 250 sampled trajectories from the posterior, and blue lines are the median trajectories. Black circles are data points that show the daily (**A**) newly reported symptomatic cases, (**B**) new hospitalizations, (**C**) current number of patients hospitalized, (**D**) current number of patients in critical care, (**E**) current number of patients undergoing mechanical ventilation, (**F**) new deaths reported, (**G**) new hospital deaths reported, i.e., excluding deaths that occurred at home or at long-term care facilities, and (**H**) number of hospital discharges. Model fits for MA and PA are shown in figs. S13 and S14.

Not all symptomatic SARS-CoV-2 infections are reported to state-level health systems. As it is difficult to make distinctions among asymptomatic, subclinical, mildly symptomatic, and symptomatic infections, here, we call an infection symptomatic if the symptoms are pronounced enough that a person with convenient zero-cost access to health care would choose to visit a hospital or clinic. Patients who cannot afford the time or cost of a health care visit are categorized as unreported symptomatic cases. Using the delays between time series of cases, hospitalizations, and deaths, we can estimate the fraction ρ of symptomatic cases that are reported to the health system (*22*, *23*). We do this without making assumptions about the CFR or IFR. Complications present themselves as underreporting in the hospitalization datasets are common (see discussions in sections S1.3 and S1.4). One clear example of this difficulty is when only current hospitalizations are available (MA and PA), a good model fit requires that the duration of hospitalization is known or identifiable; this is complicated by the fact that hospital stays come in several categories [admission to intensive care unit (ICU), requiring mechanical ventilation] and can be censored by death events. In MA and PA, there is not enough information in the remaining data streams to confidently identify the duration of hospitalization (Fig. 2B), and thus, age-stratified probability of hospital admission in MA and PA is constrained to be close to estimated values obtained from RI data (Fig. 2E). Our estimate for the reporting rate ρ in MA is 62.5% (95% CI, 54.5 to 78.5%). RI has complete reporting of hospitalization incidence, made possible by the state’s small size and a reporting system covering several small hospital networks that include all hospitals in the state. We estimate that, after May 2020, 96.3% (95% CI, 87.1 to 99.8%) of symptomatic COVID-19 cases are reported to the Rhode Island Department of Health (RIDOH). RIDOH staff and affiliated physicians reported that patients were being turned away in early March because of lack of tests, and March reporting rates are estimated at less than 30% (15 March estimate is 18.1%; 95% CI, 9.9 to 33.1%) (see Fig. 2A). For PA, our estimate of the symptomatic case reporting parameter ρ is 98.9% (95% CI, 94.5 to 99.9%), and this high rate may be the result of underreporting in multiple data streams (see Discussion). Resulting posterior model fits for RI (Fig. 3), MA (fig. S13), and PA (fig. S14) show good visual fits to the data.

**Fig. 4.**
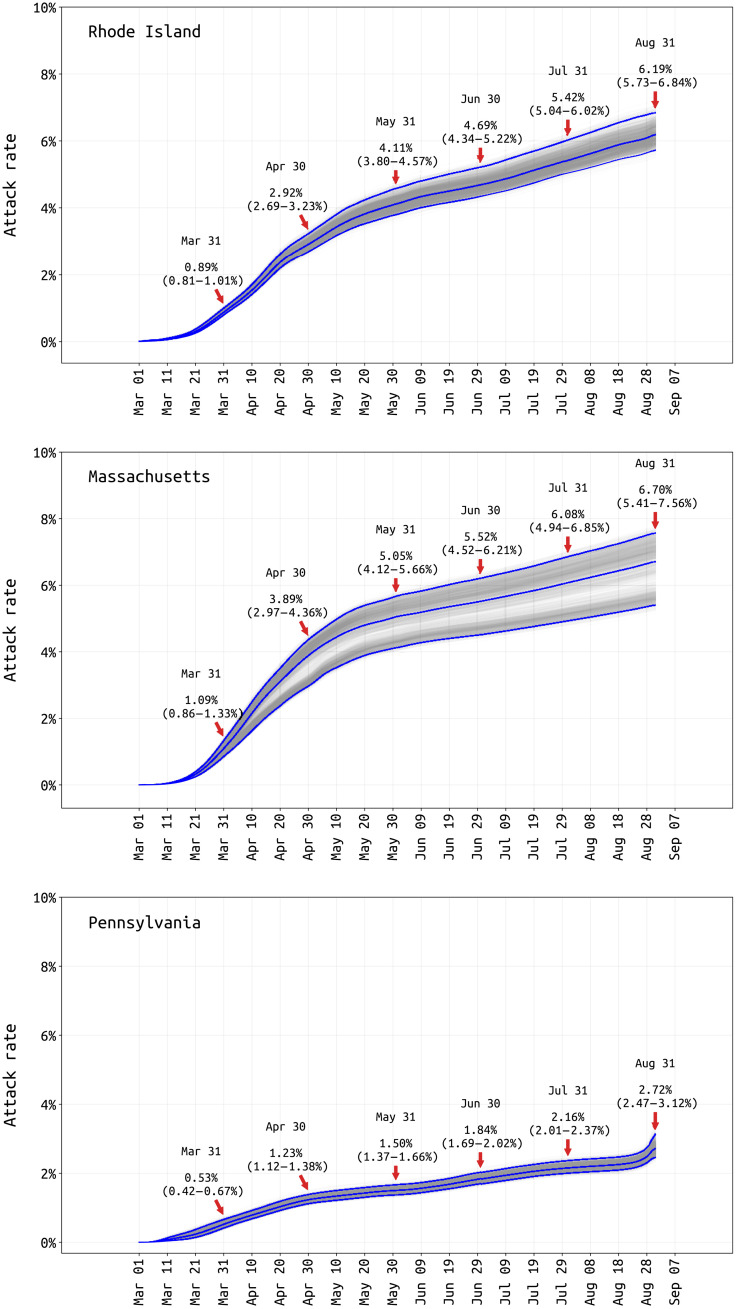
Posterior distribution of total attack rate through August 31 2020. Total infection attack rate includes all reported symptomatic cases, estimated unreported symptomatic infections, and estimated asymptomatic infections. Cumulative attack rate estimates and 95% CIs are shown for the end of every month.

Reporting rate estimates combined with age-specific estimates of asymptomatic infection (*24*) allow cumulative attack rates to be estimated (Fig. 4). The probability of asymptomatic infection is difficult to estimate for SARS-CoV-2 as this requires prospective follow-up in either a household or cohort design, with few studies including enough age groups for between-age comparisons (*25*–*28*). We use published estimates from Davies *et al.* (*24*), as the age-based asymptomatic fraction data from individual studies has too much variation to provide meaningful estimates (fig. S1). The 31 August population attack rates for SARS-CoV-2 are 6.2% in RI (95% CI, 5.7 to 6.9%), 6.7% in MA (95% CI, 5.4 to 7.6%), and 2.7% in PA (95% CI, 2.5 to 3.1%) (see Fig. 4). These attack rate estimates use symptomatic case data through 6 September, as an infection on 31 August would have its mean time of symptoms occurrence 6 days later.

Our RI attack rate can be validated with a 2.2% late-April attack-rate estimate obtained from a household serosurvey (*29*) and 0.6% early-April estimate from blood donors (*30*) (population biased toward healthier individuals). Our PA-wide attack rate has a Philadelphia early-April estimate of 3.2% as a comparator (*31*), as well as a 6.4% estimate from July using serum from dialysis patients (not adjusted for race or socioeconomic indicators and thus biased upward) (*32*). This is higher than we estimate, although we note that only 1% of Pennsylvanians had reported as symptomatic and confirmed COVID-positive through 6 September 2020; thus, the original data source may be an undercount. The unadjusted dialysis patient seroprevalence in MA was estimated to be 11.3% in July 2020 (*32*), about twice our estimate. Attack rate estimates continued to be reported to state-level DOHs through mid-March 2021 (see http://mol.ax/covid), in general agreement with studies being released during this time (see Discussion).

Estimates of reporting rates allow for age-specific fatality rate estimation in all three states (Table 1). First, these results show that the age-adjusted IFR for all three states is higher than the typically quoted 0.5 to 1.0% range over the first 8 months of IFR estimation (*33*–*37*), but note that epidemics that infect the most vulnerable segments of a population first may be associated with higher-than-average IFRs [see Discussion and (*36*, *38*)]. Population-weighted IFR estimates are 2.5% (95% CI, 2.0 to 2.8%) for the RI epidemic during March to May, 2.1% (95% CI, 1.7 to 2.5%) for the MA epidemic during March and April, and 2.8% (2.7 to 3.1%) for the PA epidemic from March to June. These estimates are presented for the early stage of each state’s epidemic as our inference suggests that mortality rates dropped from spring 2020 to summer 2020 (section S2.2), consistent with observations in New York City showing a higher than normal IFR during the first 3 months of the epidemic in 2020 (*38*). It is well known that the IFR depends strongly on age, gender, comorbidities, socioeconomic factors, and race (see Discussion) (*39*, *40*). Our estimated age-stratified IFRs indicate that fatality rates are highest (>3%) in the 60+ age groups, still very high in the 40 to 59 age group (estimates ranging from 0.3 to 1.2%), and lower in the <40 age group (<0.1%). The age-adjusted sCFR is estimated to be 3.8% (RI), 3.2% (MA), and 4.4% (PA). The hospitalization fatality rate (HFR) shows the least variation by age, with fatality rates of >9% for the >40 age groups, a lower 3.3% to 4.8% HFR for 20 to 39 age group, and no estimates possible for individuals under 20.

**Table 1.** IFR, sCFR, and HFR for the March to June COVID-19 epidemics in RI, MA, and PA. Numbers of deaths observed in the <20 age groups were too low to generate meaningful estimates.

**Age range**	**State**	**IFR (95% CI)**	**sCFR (95% CI)**	**HFR (95% CI)**
20–29	RI	<5 deaths	<5 deaths	<5 deaths
	MA	0.03% (0.03–0.04%)	0.12% (0.10–0.15%)	3.3% (2.7–4.1%)
	PA	0.04% (0.04–0.04%)	0.16% (0.14–0.17%)	4.5% (4.2–4.9%)
30–39	RI	0.05% (0.04–0.07%)	0.16% (0.13–0.21%)	3.2% (2.5–3.8%)
	MA	0.06% (0.05–0.08%)	0.19% (0.16–0.24%)	3.6% (2.9–4.4%)
	PA	0.09% (0.08–0.10%)	0.26% (0.23–0.29%)	4.8% (4.4–5.2%)
40–49	RI	0.31% (0.22–0.38%)	0.78% (0.55–0.96%)	9.6% (7.2–12.2%)
	MA	0.33% (0.26–0.44%)	0.83% (0.65–1.11%)	10.2% (7.8–12.8%)
	PA	0.58% (0.52–0.65%)	1.44% (1.31–1.63%)	17.9% (16.0–20.2%)
50–59	RI	0.60% (0.44–0.73%)	1.24% (0.90–1.49%)	10.2% (7.6–12.8%)
	MA	0.66% (0.49–0.85%)	1.34% (1.01–1.74%)	10.8% (8.2–13.5%)
	PA	1.16% (1.06–1.30%)	2.37% (2.16–2.64%)	18.8% (16.9–21.3%)
60–69	RI	3.2% (2.5–4.1%)	5.1% (3.9–6.6%)	14.0% (10.5–17.7%)
	MA	3.2% (2.4–3.9%)	5.0% (3.9– 6.2%)	14.8% (11.3–18.6%)
	PA	4.6% (4.2–5.0%)	7.3% (6.6–7.9%)	26.0% (23.2–29.4%)
70–79	RI	12.1% (9.2–13.8%)	17.5% (13.3–20.0%)	25.4% (20.4–29.9%)
	MA	10.9% (8.9–13.1%)	15.8% (12.9–19.0%)	28.2% (23.6–34.5%)
	PA	12.6% (12.0–13.4%)	18.2% (17.5–19.4%)	37.0% (34.6–40.2%)
≥80	RI	19.9% (17.1–23.5%)	28.9% (24.8–34.0%)	29.7% (24.4–34.5%)
	MA	19.2% (16.3–22.9%)	27.8% (23.7–33.2%)	32.8% (27.8–39.6%)
	PA	21.7% (20.6–23.5%)	31.5% (29.8–34.1%)	42.4% (39.7–45.8%)

### Changes in age-stratified contact patterns and clinical outcomes during the epidemic

**Fig. 5.**
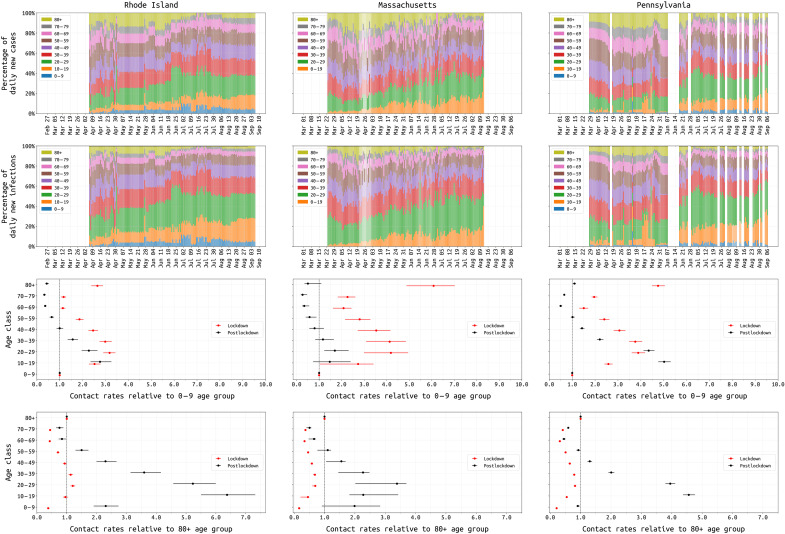
Changing age-structure of COVID-19 epidemics in RI, MA, and PA. Top rows show the age structure of reported cases (first row) and estimated infections (second row) from 1 March to 31 August. RI and PA report age data periodically; missing values have been linearly interpolated in RI. Third row shows the inferred age-specific contact rates (median and 95% CIs) for both the lockdown (red) and postlockdown period (black), where the reference group is the 0 to 9 age group. Fourth row shows these same inferred contact rates with the ≥80 age group as the reference.

We investigated changing age-specific contact rates during the three state epidemics, based on observed changes in age distribution and well-documented reporting of outbreaks in nursing homes. In 2020, age-contact matrices began to be measured for the COVID-19 socially distanced era (*41*–*45*), and we thus allow for two mixing patterns in our population—one mixing pattern during the spring 2020 lockdown and a second pattern in late spring and summer after the lockdowns were lifted. We infer eight relative mixing levels for each age class (relative to the 0 to 9 age group) and use a symmetric parametrization where contact rates are described per age group pair (i.e., *c_ab_* = *c_a_* × *c_b_*) where *c_a_* is the mixing rate for age group *a*; see equations (section S6). Use of the Belgian CoMix study’s contact rates was evaluated (*43*, *46*), but these more highly parameterized matrices did not provide a better fit for our 6-month time series [ΔDIC (deviance information criterion) > 70 for all three states]. Age characteristics of each state epidemic are shown in Fig. 5 (top rows), and the inferred contact parameters are shown in Fig. 5 (bottom rows); inference of contact rates is influenced by the model assumption that the 0 to 19 age group is 60% as susceptible to infection as the other age groups (*24*). In all three states, the lowest inferred contact rates during lockdown were for the 0 to 9 and 60 to 79 age groups, reflecting closed schools and possibly the caution with which older individuals approached their risk of infection. However, the relative contact rates for individuals in the ≥80 age group were much higher: 2.6 (95% CI, 2.4 to 2.9) in RI, 6.1 (95% CI, 4.9 to 7.0) in MA, and 4.8 (95% CI, 4.5 to 5.1) in PA. This suggests that social distancing and lockdown were more difficult for individuals that needed additional care or lived in congregate care facilities. The shift from an older age profile to a younger age profile is apparent in all three states’ epidemics as the epidemics progressed from spring to summer (Fig. 5, top rows).

Improvements in clinical management of hospitalized COVID-19 cases, due to the use of prone positioning (*47*, *48*) or more frequent use of corticosteroids (*49*, *50*), may have led to lower mortality relative to epidemic size during the more recent (June to August) stages of the epidemic when compared to March to May mortality rates (*51*–*53*). To estimate the effects of some of these interventions, we assess whether progression from hospitalization to critical care changed between the early stages and the later stages of the epidemic. Our model uses the relative age proportions described by Lewnard *et al.* (*54*) who estimated probabilities of progression from medical floor care to critical care to be between 30 and 50% [comparable to other estimates (*55*, *56*)] for all nine age bands used in this study. These age-specific probabilities are scaled in our model (keeping the relative age probabilities the same), independently for each state, as patterns of hospital admission and clinical algorithms for ICU admission are likely to differ somewhat between health systems and hospitals; the scaling parameter is estimated. In RI, the age-adjusted probability (posterior median) of ICU admission for a hospitalized case dropped from 26.0% (95% CI, 20.4 to 31.0%) to 16.5% (95% CI, 11.6 to 21.0%) with an inferred breakpoint at 26 May 2020 (95% CI, May 6 to June 2). In PA, the age-adjusted ICU admission probability dropped from 39.5% (95% CI, 36.7 to 43.1%) to 28.0% (95% CI, 24.4 to 33.8%), with an inferred breakpoint of June 19 (95% CI, June 13 to June 22). In MA, this probability dropped from 29.2% (95% CI, 23.9 to 36.3%) to 21.2% (95% CI, 17.5 to 29.0%), with the likely change occurring between late April and mid-June (Fig. 2D).

A second approach to confirming trends on improved clinical case management would be to look directly at changes in mortality. However, the complexity in this analysis lies in the different possible clinical paths that lead to a fatal outcome. In most states, reported mortality trends combine deaths occurring in hospitals with deaths occurring outside hospital settings (i.e., in congregate care facilities); these data streams are separated in RI/PA but not MA. Our model allows for inference of mortality outside hospital settings, with the outside-hospital sCFR estimated at between 20 and 35% for the ≥80 group and 5 and 20% for the 70 to 79 age group (Fig. 2C). This allows us to separate mortality trends between in and out of hospital, but hospital mortality alone is a complex composite of probability of death on the medical floor level of care and probability of death in the ICU (with and without ventilation). For this reason, we chose ICU admission as the clinical progression marker where we could evaluate a simple trend of improved case management. Estimated outside-hospital mortality may be affected by the choice of using reported death counts or excess death counts (*57*) in an analysis, and using excess deaths in our inference did result in slightly higher estimates of outside-hospital mortality for the >70 age groups (section S6). Model selection analyses showed that a model with changing age-mixing patterns and improved clinical management was a better fit than models without these features (ΔDIC > 27 for all three states; Table 2).

**Table 2.** DIC values for different models. Minimum DIC values shown in boldface.

	**No change** **in age** **profile of** **contact** **rates after** **lockdown.**	**Different** **age** **profiles of** **population** **contact** **rates after** **lockdown** **(approx.** **May 2020)**	**No change** **in age** **profile of** **contact** **rates after** **lockdown.**	**Different** **age** **profiles of** **population** **contact** **rates after** **lockdown** **(approx.** **May 2020).**
	**No change** **in ICU** **admission** **rate from** **March to** **August**	**No change** **in ICU** **admission** **rate from** **March to** **August**	**Allows for** **change in** **ICU ** **admission** **rate from** **March to** **August**	**Allows for** **change in** **ICU ** **admission** **rate from** **March to** **August**
RI	12,064	11,059	11,960	**11,004**
MA	26,123	18,710	26,328	**17,603**
PA	18,858	10,626	18,851	**10,599**

## DISCUSSION

This is among the first studies to evaluate multiple simultaneous clinical data streams with an epidemic transmission model. The analysis of concurrent data streams is necessary to describe certain important but unreported characteristics of regional SARS-CoV-2 epidemics; these include underreporting of cases, changing age patterns of infection, changing patterns of clinical progression, and an understanding of mortality rates outside hospital settings. The inclusion of multiple age-structured data streams on death and hospitalization allows for statistical estimation of symptomatic case underreporting—a quantity that is generally resistant to robust estimation especially in public health reporting systems that (i) mix active and passive surveillance, (ii) mix multiple diagnostic tests and testing visits, and (iii) have not made estimates of their catchment areas. With an estimate of symptomatic case underreporting (here, via ρ), we can estimate the population-level SARS-CoV-2 attack rate in each state by summing the reported symptomatics, the unreported symptomatics, and an externally estimated number of asymptomatics. One month later, an attack rate estimate can be validated by comparing to results from a seroprevalence survey. Four seroprevalence estimates available for RI, MA, and PA show no major inconsistencies with our results. It is important to remember that SARS-CoV-2 serosurveys can be subject to biases depending on approaches to recruitment (which can overestimate seropositivity if enriched for individuals who are more likely to have been infected, e.g., individuals who consent because of past symptoms), the time since the original infection (antibody titers wane over time), and the specific test used (*58*).

Our results indicate that in autumn 2020, RI, MA, and PA were nearly fully susceptible to a winter epidemic wave of SARS-CoV-2. Continual attack-rate estimation is crucial for epidemics in naïve populations to assess remaining susceptibility. Specifically, real-time age-specific attack rate estimation is important for vaccination planning, as age groups experiencing the least infection may need to be prioritized during both initial and annual vaccination campaigns. Including regular preplanned population-representative serosurveys (*59*–*62*) as part of routine public health activity (*63*, *64*) would provide substantial benefit in being able to assess the progress and trajectory of an ongoing epidemic.

Similar to attack-rate estimation, mobility tracking can give us a partial window into the effect that distancing policies or lockdowns are likely to have on viral transmission. In May 2020, the positive correlation between stay-at-home metrics and viral transmission vanished in all three states [as in (*65*)], resulting in a summer with population mixing levels at nearly prepandemic levels (i.e., people not staying at home) but viral transmission close to its postlockdown low point. It is reasonable to suggest that some of this is explained by (i) weather increasing the proportion of contacts made outdoors, where transmission is known to be much less likely, and (ii) a shift from mixing outside the home to inside the home, i.e., less time spent at work and more time with family. It is not straightforward to relate measures of population movement to opportunities for transmission, for many reasons including the collinearity of mixing with many other factors that can influence it. Essentially, rather than absolute measures of mixing, the blue lines in Fig. 1 can be interpreted as levels of population mixing that are capable of producing transmission (“transmission-capable mixing”). By canceling large events, promoting stringent hygiene measures, requiring masking, closing schools, restricting gathering sizes, and creating new guidelines for business operations, the epidemics in RI, MA, and PA were contained during the summer months while allowing the states’ residents to continue most essential activities including small/medium outdoor events. In summer 2020, aggregate measures of population movement were at or near normal levels, but mixing leading to transmission was substantially reduced.

### Comparison of attack rate estimates

It is useful to compare our results on attack rate and contact patterns with those obtained through different methodological approaches. Our U.S. state-level inference was performed on a data stream of cases, hospitalizations, and deaths, with an externally estimated asymptomatic fraction; we estimated mixing/mobility levels, underreporting for symptomatics, and the IFR. The state-level analyses presented by Unwin *et al.* (*66*) and Monod *et al.* (*67*) performed inference on a data stream of cases, mobility, and deaths, with an externally estimated IFR; they estimated age-pair contact rates and underreporting for infections. Certainly, both approaches’ results are sensitive to the external estimates used. The combined effect of underreporting and asymptomatic infection (Unwin’s infection ascertainment ratio defined as the number of reported cases divided by the total number of infections) has similar estimates whether using our approach or Unwin’s—0.58 (here) and 0.51 (Unwin) in RI, 0.43 and 0.38 in MA, and 0.59 and 0.51 in PA. However, the 1 June 2020 attack rates estimated with these two approaches differ by factors of two or three—4.1% (here) and 7.5% (Unwin) in RI, 5.0 and 11.2% in MA, and 1.5 and 4.4% in PA—suggesting that the external estimates of IFR and the asymptomatic fraction play a large role in attack rate estimation. Comparing our attack-rate estimates to those of Monod (through late Oct 2020), we see estimates of 10.9% [our method (*68*)] and 11.0% [Monod *et al.* (*67*)] in RI, 8.2 and 13.0% in MA, and 6.8 and 6.6% in PA. For MA and RI, these late-October estimates are not consistent with U.S. Centers for Disease Control and Prevention (CDC)’s commercial laboratory seroprevalence survey (*63*) (about 5% in RI, 4% in MA, and 7% in PA), or the CDC blood donor survey (*64*) seroprevalence estimates (4% in RI, 5% in eastern MA, and 5% in central/southwestern PA). The CDC results need to be evaluated in the context of rising and waning seroprevalence, which may result from a high assay threshold and/or not accounting for antibody waning in seroprevalence estimates (*69*, *70*).

Our data and inference support a changing contact pattern in May/June 2020, with much higher mixing levels for the 10 to 29 age group in summer than in spring. This contact pattern was not explicitly tested by Monod *et al.* (*67*), although they did find that the 20 to 49 age group was the primary driver, at a national scale, of transmission in summer and fall 2020; the influence of the 20 to 49 age group on transmission appears to be small in RI, MA, and PA as these states did not have rebound epidemics in summer 2020. Despite differing approaches as to which quantities are treated as data and which ones are estimated, our study and Monod’s share a point of consistency in the importance of the 20 to 29 age group to maintaining transmission in summer 2020.

### Comparison of fatality rate estimates

In our analysis, IFRs are estimated to be higher than in recently summarized analyses (*33*–*37*), and the differences are particularly notable in the 50 to 79 age group where we infer IFRs that are 1.5 to 2.5 times higher than previous estimates. Our IFR estimates for the 20 to 49 age groups are most similar to those presented by Brazeau (*37*), and the all-ages IFRs in MA and RI are as high as some of the highest estimates (*9*) of the study of Levin *et al.* (*34*). These high estimates may correspond to a high degree of exposure heterogeneity in the studied epidemics. As it is known that RI and MA had substantial outbreaks in elderly care facilities in the spring, it is likely that this focused epidemic passed through a more susceptible subpopulation (individuals who cannot fully quarantine or distance because of needing routine care) that is also more likely to progress to severe clinical outcomes including death. This was observed in New York City, where an infection-weighted IFR of 1.39% was estimated for the first several months of the epidemic in 2020 (*38*). When weighting our estimates by the number of infections in each age class (this is a particular epidemic’s IFR as opposed to the IFR for a randomly selected person in the population), we obtain IFRs of 2.24% for RI and 1.53% for MA for spring 2020.

A second possible explanation for the high estimates of IFR and sCFR presented here is that RI, MA, and PA reported COVID-19 death counts similar to excess death counts for the same period. This implies that in locations where deaths were undercounted, the excess death counts are closer to the true COVID-19 death counts. A third possibility (for PA only) that would influence both IFR and attack-rate estimates is that both case and hospitalization data were underreported. A 1% symptomatic attack rate in PA over the first 6 months of the pandemic is lower than expected, but the underreporting fraction estimate (ρ = 98.9%) in our analysis is likely wrong if hospitalization numbers were also underreported. The reporting rate ρ determines the attack-rate estimates; if ρ is overestimated, then the IFRs presented here would also be overestimated. This may be the reason that our estimated fatality rates for PA appear to be unreasonably high and that the PA attack-rate estimate through 31 August 2020 is so low.

The sCFRs inferred for RI, PA, and MA (estimates range from 3.2 and 4.4%) are in the higher ranges of previously reported estimates (*7*–*11*, *71*, *72*), suggesting that the individuals infected during the spring wave and summer lull were more likely to progress to symptoms than the average person in the population. Again, this is consistent with the observation that children were the least exposed in the spring and summer months, and thus, the exposed population was both more likely to progress to reportable symptoms and more likely to progress to severe clinical outcomes.

In late 2020, a counterproductive diversion in policy discussion was the consideration of an epidemic management approach that would encourage younger/healthier populations to become infected (*73*). Our state-level analyses indicate that older individuals are not able to fully isolate during lockdown periods. This makes a “protecting the vulnerable” strategy unworkable, as vulnerable individuals will still require essential care and contact with other humans. Any policy aiming to protect vulnerable individuals while allowing the remainder of the population to mix and move freely would almost certainly fail at preventing viral introduction from the general population into vulnerable populations (*74*). In our analysis, during the March/April lockdown period, the ≥80 contact rate was the highest or among the highest when comparing across age groups (Fig. 5). As individuals in the oldest age groups are relatively unaffected by lockdown, the best way to protect these (and other) vulnerable populations is to limit the spread in the general population.

### Limitations and recommendations

One key limitation in using data streams rooted in symptomatic case reporting is the inability to infer asymptomatic infection rates. These rates must be estimated independently from cohort follow-up or contact tracing. They are susceptible to bias in the younger age groups if children test negative because of low viral loads and are classified as negative rather than asymptomatic. Studies are also susceptible to design errors when the protocol or data collection does not allow for differentiation between presymptomatic and asymptomatic individuals (section S1.7). Although most studies have converged on an age-adjusted “60% symptomatic” number, age-specific estimates come with less certainty and differences in diagnostic tests and testing protocols have resulted in substantial variation in these estimates (fig. S1).

The data streams we present here do not allow us to evaluate the degree to which the epidemic runs through specific subpopulations (e.g., congregate care settings and college students) that are more vulnerable, susceptible, or transmit more easily. To measure variability in transmission and susceptibility from state-level data, we suggest including these common data types into the same databases/datasheets currently maintained by all state DOHs as part of routine COVID reporting: (i) contact counts and positivity rates from contact tracing efforts, (ii) positive/negative case counts and inclusion criteria from asymptomatic random screening programs (*75*), and (iii) a datasheet keyed on a categorical variable of “infection source event” with confirmed patient counts, ages, and dates of reporting listed (*76*, *77*). Among these data types, the asymptomatic screening efforts are likely the easiest to turn into a standardized daily data stream as samples taken from screening programs pass through the same sample/data processing pipelines as samples from symptomatic patients. These data would also allow for real-time tracking of prevalence (*78*).

We cannot exclude the possibility that our reporting rate estimate (ρ) is incorrectly estimated because of model misspecification or data integrity problems. In addition, symptomatic reporting is likely to vary by age (*38*) and by availability of testing, making a single ρ estimate a coarse descriptor of individuals’ reporting tendencies. This is the reason that validation with seroprevalence estimates is crucial for estimating underreporting in public health surveillance systems. The entire inferential framework for ρ assumes that hospitalization data are complete, that death data are complete, and that various measures of hospitalization duration have been independently estimated or are identifiable from our data. The biggest leap in these assumptions comes in the completeness of hospitalization data, as both MA and PA have relied on hospitalization data streams that are partially complete. This is a reminder, during the pursuit of rapid results with prepackaged epidemiological tools and dashboards, to carry out the somewhat slower due diligence of understanding the sampling frames of all data streams included in an analysis. If hospitalization numbers are underreported in other states as well, then national-level analyses of hospitalization numbers would need to acknowledge and account for this.

Last, we were not able to use any published contact matrices for the lockdown period, as these data did not exist for our populations at the time our work was being done (*41*–*45*). Thus, we used nine independent mixing rates for the nine age classes in our model (and assumed that contact between two age groups is proportional to their two mixing rates); the data are unlikely to have enough resolution to infer 81 independent mixing parameters.

Through 2019, infectious disease epidemiology was neglected in the United States for more than half a century because of our status as a developed country with a secure food supply, a sanitary water system, few persistent disease vectors, high public hygiene standards, and ample supply of therapeutics and vaccines. We were not prepared in 1981 when the HIV epidemic was uncovered, and with no leadership from the federal government in 2020, we were underprepared at the state level for the SARS-CoV-2 epidemic, as few individuals remained with knowledge from the early struggle against HIV. Specifically, the right data systems were not in place at state-level DOHs to provide consistent and interpretable data streams allowing epidemiologists to make real-time assessments on epidemic progression and success of control efforts. State-level systems in the United States require more funding from the federal government or centrally designed (and funded) reporting tools from the Centers from Disease Control and Prevention that would allow all states to consistently report the same high-quality data types. The rationale for this systems upgrade would be to advance our surveillance systems to those of countries such as New Zealand, Hong Kong, Singapore, Vietnam, Taiwan, and South Korea that successfully controlled epidemic waves and introductions of SARS-CoV-2.

## METHODS

### Case data

Eleven data streams were assembled from three state DOH websites and data dashboards: (I) cumulative confirmed cases, (II) cumulative confirmed cases by age, (III) cumulative hospitalized cases, (IV) cumulative hospitalized cases by age, (V) number of patients currently hospitalized, (VI) number of patients in ICU currently, (VII) number of patients on mechanical ventilation currently, (VIII) cumulative deaths, (IX) cumulative deaths by age, (X) cumulative hospital deaths, and (XI) cumulative hospital discharges. Data streams VI and XI were missing in PA, and X and XI were missing in MA. Cumulative hospitalizations (data streams III and IV) in MA and PA were reported as a subset of symptomatic cases (via follow-up case investigations) and were excluded from the analysis. Reporting started on 27 February (RI), 1 March (MA), and 6 March (PA) and datasets used in this analysis comprise about 180 days of data through 6 September. Age-specific counts often summed up to be less than the corresponding total daily counts of new symptomatic cases, new hospitalizations, or new deaths. This was common because of lack of age reporting in some proportion of cases. We assumed missing age-structured data to be missing completely at random when their sum was less than the total count.

### Mobility data

The first set of mobility data is provided by the COVID-19 Mobility Network (*21*) and is derived from users of the Facebook mobile app with the location history option enabled, representing approximately 0.8% of MA, 1.1% of PA, and 1.1% of RI. Each user’s location is binned into tiles, approximately 470 m by 610 m at PA’s latitude. These are aggregated by home county and date and reported as the fraction of users who remain in one tile for the whole day. Here, we report state-level data by weighting these proportions by each county’s population, per the U.S. Census’ 2019 estimates. These estimates are not adjusted for the demographics of Facebook’s user base.

The second set of mobility data is provided by social distancing metrics recorded by SafeGraph (*79*). The data were derived from Global Positioning System (GPS) pings of anonymous mobile devices. A common nighttime location for each device over a 6-week period was defined to be the device’s “home,” and daily GPS pings were analyzed to determine whether the device exhibited certain behaviors including completely staying at home, working part time, working full time, etc. The counts were aggregated at the Census Block Group (CBG) level, which is the second-smallest geographical unit for which the U.S. Census Bureau publishes data. A state-level percentage at home fraction can be calculated by dividing the “completely at home” devices in a state by the total devices in that state; however, one step was taken before this calculation as outlined in the data analysis methodology for the Stay-At-Home Index provided by SafeGraph (*80*). The step included was a correction for sampling bias at the CBG level by resampling with a stratified reweighting method described in section S1.5.2 (*81*).

### Mathematical model

A standard age-structured ODE model was used to describe the dynamics of SARS-CoV-2 spread in a single well-mixed population. The model includes 30 compartments for different clinical states including susceptible, exposed, asymptomatic, infected, hospitalized, in ICU, and on mechanical ventilation. Multiple consecutive compartments are used for most clinical states to reduce the variance on length-of-stay in various stages in disease progression. Model diagram is shown as fig. S2, and equations are shown in section S2.

Model parameters fall into several categories including parameters on contact rates, lengths of stay in various clinical states, and probabilities of progression from one state to another. Daily community-level transmission-capable mixing rates β_t_ were inferred from the data, while age-specific contact rates for hospitalized individuals had to be fixed as too little data exist on these parameters. Asymptomatic individuals are assumed to be half as infectious as symptomatic individuals [similar to other models’ assumptions (*82*, *83*)].

Lengths of stay and age-specific probabilities of clinical progression were available from numerous datasets documenting COVID-19 hospitalized populations (details in section S2, table S1 and https://github.com/bonilab/public-covid19model). When clinical parameters were inferred, their median estimates were typically close to observed values in hospital or surveillance datasets. No data were available in RI, MA, or PA to infer the asymptomatic fraction for each age group, and these were obtained from cohort analyses available at the time (see section S1.7) and the inferred age-specific asymptomatic fractions in Davies *et al.* (*24*); the Davies fractions were used for the final model runs.

### Statistical inference

Given the various, and at times incomplete, data sources available for each state, we chose a flexible Poisson-Gamma process–based likelihood framework to facilitate inference of ODE model parameters while accounting for model uncertainty. In particular, the cumulative cases, hospitalizations, deaths, and hospital discharge data were assumed to be realizations of conditionally independent, inhomogeneous negative binomial processes, with time-varying process rates defined by the expected deterministic ODE output. The likelihood function for each age-structured data stream is then a product of independent, negative binomial increments, with means determined by the corresponding age-structured component of the ODE system over each increment. Means for observed new symptomatic cases were equal to ODE system predictions multiplied by a symptomatic reporting rate constant, and means for observed new hospitalized individuals were equal to ODE system predictions. The time from symptoms to presentation was fixed at 2.0 days (see section S5.2). Dependence across data streams is assumed to be captured by the ODE system. Total data streams, summed over all age classes, were viewed as the sum of independent negative binomial random variables and are as such negative binomial random variables themselves, with mean given by the sum of the age-specific means. When both age-structured and total data are available, we assume that any missing age-structured data are missing completely at random and approximate the joint likelihood of the total and age-structured counts by ignoring overdispersion and assuming that, conditioned on the total data, the age-structured counts are multinomially distributed with probabilities proportional to the age-structured ODE means. Data on current hospitalizations, as well as current numbers in ICUs, and current intubated individuals were modeled using reported weekly totals. The total number of intubated individuals, individuals in ICUs (but not intubated), and hospitalized individuals (not in ICUs) was each modeled as independent Gaussian random variables with means equal to the corresponding totals predicted by the ODE system and with unknown variances. Additional details on the likelihood framework can be found in section S3.

We chose a Bayesian approach to inference, allowing for appropriate penalization of time-varying parameters and a combination of strongly and weakly informative priors for parameters relating to clinical progression of disease. The composite rate parameter, β_t_, describing person-to-person contact mixing, is constructed via a cubic B-spline, with a random walk prior (penalized regression spline with first order differences) on the B-spline coefficients to penalize overfitting. In RI and PA, the symptomatic reporting rate ρ is constructed as an I-spline with a similar prior; in MA, it is assumed to be constant across time. Additional parameters found within the ODE system, including length of hospital stay and proportion of cases needing hospitalization within each age class, are given uniform priors with bounds determined by expert judgement, while negative binomial dispersion parameters are given weakly informative exponential priors and Gaussian variance parameters are given conjugate inverse-gamma priors. Given these priors and the previously defined likelihood, we constructed a Markov chain Monte Carlo algorithm to sample from the posterior distribution of model parameters. Block updates for parameters were obtained using a random walk Metropolis-Hastings algorithm with an adaptive proposal distribution (*84*). For each state, five independent chains were run for 300,000 iterations, with the first 100,000 samples discarded as burn-in. Convergence was assessed qualitatively across the five chains. R and C++ code are posted at https://github.com/bonilab/public-covid19model.

## Supplementary Materials

### This PDF file includes:

Sections S1 to S7 Table S1 Figs. S1 to S17 References

## Notes

View/request a protocol for this paper from *Bio-protocol*.
